# Supplementary material for: BcWRKY33A Enhances Resistance to Botrytis cinerea via Activating BcMYB51-3 in Non-Heading Chinese Cabbage
Source: Int J Mol Sci. 2022 Jul 26;23(15):8222. doi: 10.3390/ijms23158222 (PMC9331318; doi:10.3390/ijms23158222)
Supplement: Supplementary file 1 [file ijms-23-08222-s001.zip › ijms-1812212-supplementary.pdf]

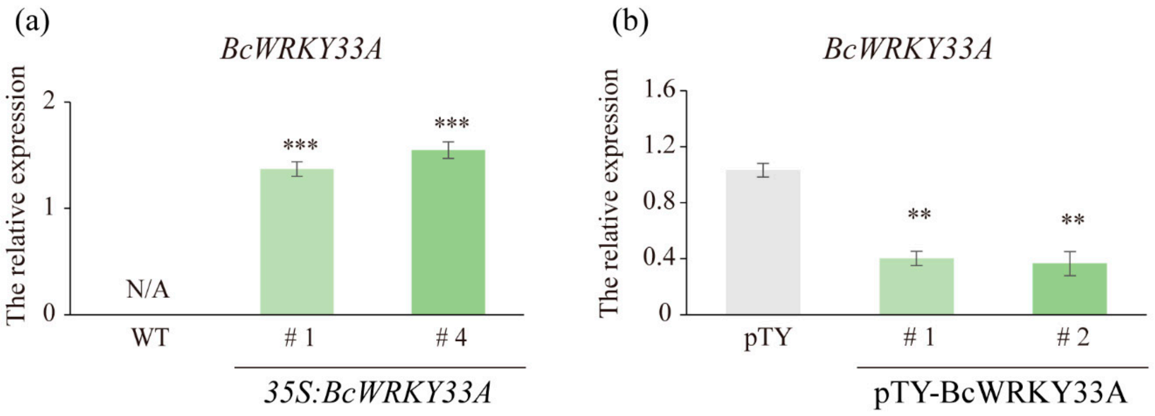

Figure S1 Establishment of *BcWRKY33A*-overexpressed and silenced lines.

(a) The relative expression of *BcWRKY33A* in WT and *35S:BcWRKY33A* lines. N/A represents no expression. (b) The relative expression of *BcWRKY33A* in pTY and pTY-BcWRKY33A. Error bars represent standard deviation (±SD). The data are the mean ± SD of three biological replicates. Asterisks indicate significant differences from the control (**$p < 0.01$, ***$p < 0.001$) using Student's *t*-test.

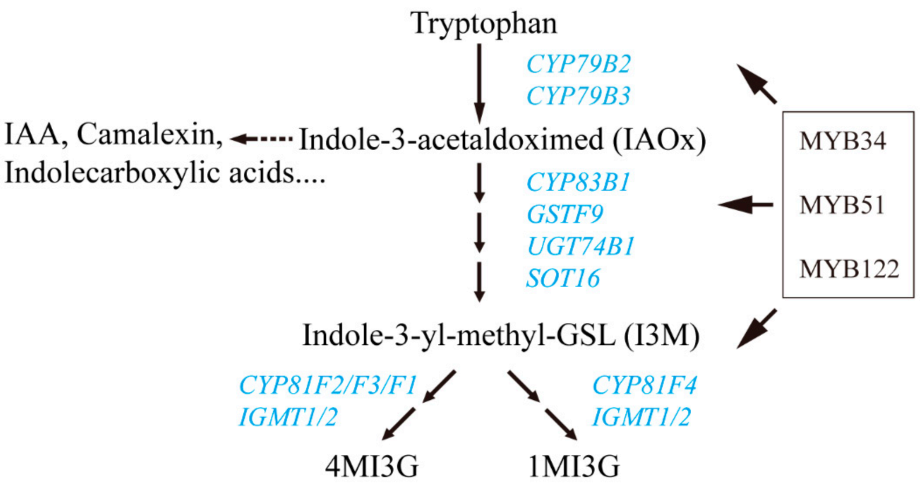

Figure S2 Schematic plot of the IGSs biosynthetic pathway.

Figure S2 is adapted from a previous study (Frerigmann & Gigolashvili, 2014), in *Arabidopsis*, IAOx was not only needed for the formation of IGSs, but also the precursor of indole-3-acetic acid (IAA), camalexin and indole-carboxylic acids, etc. (indicated by dotted arrow). Enzymatic steps are indicated by black arrows. 4MI3G, 4-methoxyindole-3-ylmethyl glucosinolate; 1MI3G, 1-methoxyindole-3-ylmethyl glucosinolate.

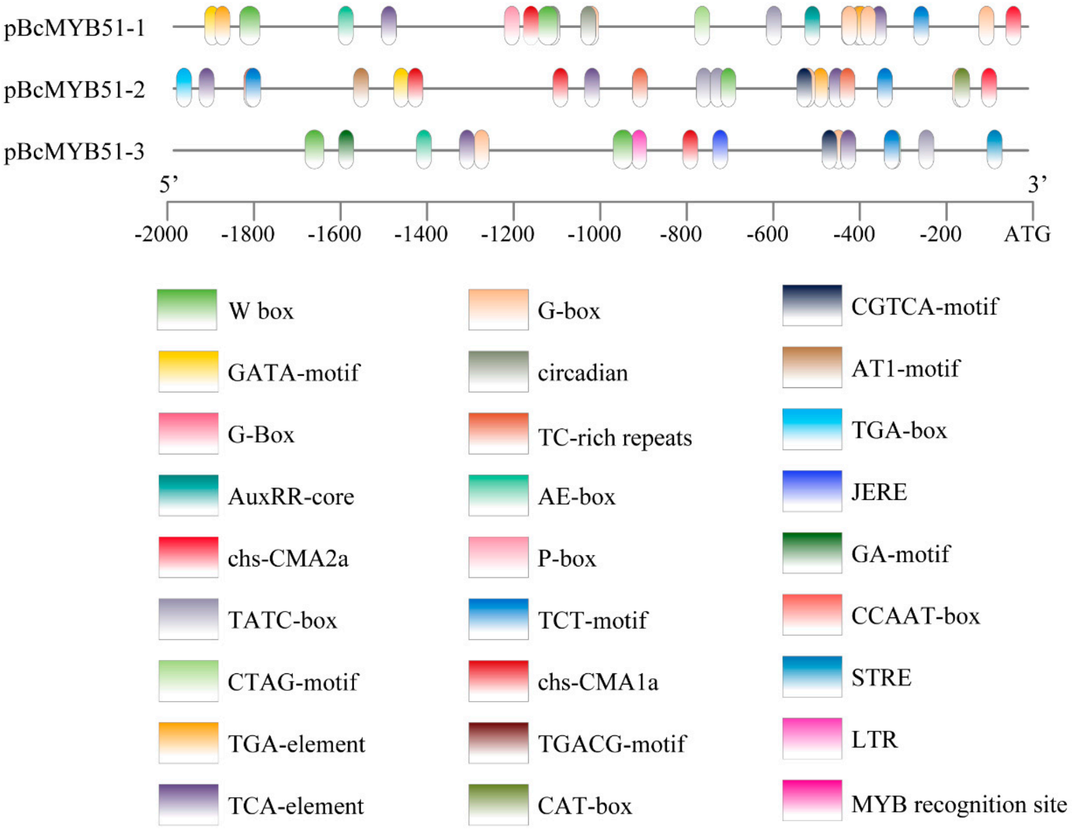

Figure S3 The *cis*-elements distribution in the promoters of *BcMYB51s*.

pBcMYB51-1, pBcMYB51-2, pBcMYB51-3 represent the promoter of BcMYB51-1, BcMYB51-2, BcMYB51-3, respectively.

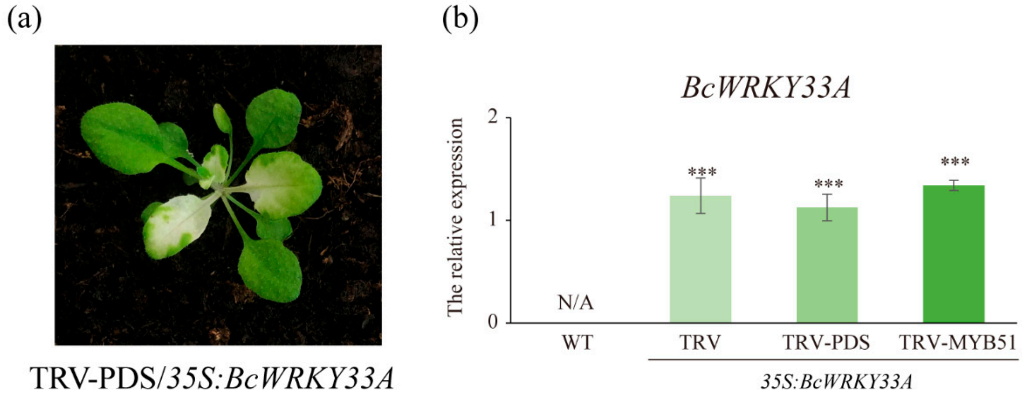

Figure S4 The efficient TRV-based VIGS system.

(a) The phenotype of TRV-PDS/*35S:BcWRKY33A* lines. (b) The relative expression level of *BcWRKY33A* in WT, TRV/*35S:BcWRKY33A*, TRV-PDS/*35S:BcWRKY33A*, TRV-MYB51/*35S:BcWRKY33A* lines. N/A represents no expression. Error bars represent SD. Data are means ± SD of four biological replicates. Asterisks indicate significant differences from the WT (***$p < 0.001$) using Student's *t*-test.

Table S1 The function prediction of *cis*-elements in promoters of *BcMYB51s*.

***cis*-elements**	**function prediction**
AE-box	part of a module for light response
AT1-motif	part of a light responsive module
AuxRR-core	cis-acting regulatory element involved in auxin responsiveness
CAT-box	cis-acting regulatory element related to meristem expression
CCAAT-box	CCAAT-box
CGTCA-motif	cis-acting regulatory element involved in the MeJA-responsiveness
chs-CMA1a	part of a light responsive element
chs-CMA2a	part of a light responsive element
circadian	cis-acting regulatory element involved in circadian control
CTAG-motif	CTAG-motif
GA-motif	part of a light responsive element
GATA-motif	part of a light responsive element
G-Box	cis-acting regulatory element involved in light responsiveness
JERE	JERE
LTR	cis-acting element involved in low-temperature responsiveness
MYB recognition site	MYB recognition site
P-box	gibberellin-responsive element
STRE	STRE
TATC-box	cis-acting element involved in gibberellin-responsiveness
TCA-element	cis-acting element involved in salicylic acid responsiveness
TC-rich repeats	cis-acting element involved in defense and stress responsiveness
TCT-motif	part of a light responsive element
TGA-box	part of an auxin-responsive element
TGACG-motif	cis-acting regulatory element involved in the MeJA-responsiveness
TGA-element	auxin-responsive element
W box	W box

Table S2 The sequence of probes used in EMSA

Name of probes	Sequence	Note
Probe 1	ATGTTGACTAGTTATGTTGACTAGTT	biotin labeled
Competitor 1	ATGTTGACTAGTTATGTTGACTAGTT	no labeled
Probe 2	AGAAAATTCAACGGAGAAAATTCAACGGAGAAAATTCAACGG	biotin labeled
Competitor 2	AGAAAATTCAACGGAGAAAATTCAACGGAGAAAATTCAACGG	no labeled

Table S3 Primers used in this study

Table S3 Primers used in this study.		
**Primer Name**	**Sequences (5'-3')**	**Function**
BcMYB51-1F	CAACCCCATCAAGAATCAAG	**Cloning**
BcMYB51-1R	TCATGCAAAATAGTTATCAAC	
pBcMYB51-1F	GAACAGATTAAAAACAGAATG	
pBcMYB51-1R	TGTCCGCACCATTCTTGATTC	
BcMYB51-2F	ATGGTGCGGACACCATGTTGCAA	
BcMYB51-2R	TGTGGGATTCGTCAGAAGATTCACGC	
pBcMYB51-2F	GACGTAAACAAAATTGTGAAG	
pBcMYB51-2R	TGTCCGCACCATTCTTGATTC	
BcMYB51-3F	TCGAATCACTAGCTCAAAAAAGCTGAGA	
BcMYB51-3R	TCTTCAAAACTGTCTTGATGCTC	
pBcMYB51-3F	GATTTAGTTGTCTTCTTTTAGCAT	
pBcMYB51-3R	TGTCCGCACCATTCTTGATTC	
PDS-F	TTCTGCGGCGAATTTGCCTTATCAAAACG	
PDS-R	AGAAACTCTTAACCGTGCCATCGTCATTGAG	
MYB51-F	AGCTCGTGGACTACCAGGAA	
MYB51-R	TTGACGTTCATAGACCGGCG	
pRI101-BcMYB51-1F	TCTTCACTGTTGATACATATGATGGTGCGGACACCG	**Subcellular localization**
pRI101-BcMYB51-1R	GCTCACCATGGATCCGGTACCTGCAAAATAGTTATC	
pRI101-BcMYB51-2F	TCTTCACTGTTGATACATATGATGGTGCGGACACCA	
pRI101-BcMYB51-2R	GCTCACCATGGATCCGGTACCTGTGGGATTCGTCAG	
pRI101-BcMYB51-3F	TCTTCACTGTTGATACATATGATGGTGCGGACACCA	
pRI101-BcMYB51-3R	CATGGATCCGGTACCTGCAAAATAGTTATCAATA	
pABAi-pBcMYB51-1F	GAAAAGCTTGAATTCGAGCTCGAACAGATTAAAAAC	**Yeast-one-hybrid assay**
pABAi-pBcMYB51-1R	AGCACATGCCTCGAGGTCGACTTGAGGATGAGGCAC	
pABAi-pBcMYB51-2F	GAAAAGCTTGAATTCGAGCTCGACGTAAACAAAATT	
pABAi-pBcMYB51-2R	AGCACATGCCTCGAGGTCGACTGTGGTGGAGGCACC	
pABAi-pBcMYB51-3F	GAAAAGCTTGAATTCGAGCTCGATTTAGTTGTCTTC	
pABAi-pBcMYB51-3R	AGCACATGCCTCGAGGTCGACTTGAGGAAGAGGCAC	
nBcWRKY33A-AD-F	GCCATGGAGGCCAGTGAATTCATGGCTGCTTCTTCT	
nBcWRKY33A-AD-R	CAGCTCGAGCTCGATGGATCCTCACCTTCCATTGTA	
cBcWRKY33A-AD-F	GCCATGGAGGCCAGTGAATTCATGGAGCAAAGGAAA	
cBcWRKY33A-AD-R	CAGCTCGAGCTCGATGGATCCTCAGGACAAAAACGA	
p0800-pBcMYB51-3F	CTTGATATCGAATTCCTGCAGGATTTAGTTGTCTTC	**Dual-luciferase reporter assay**
p0800-pBcMYB51-3R	CGCTCTAGAACTAGTGGATCCTTGAGGAAGAGGCAC	
pRI101-BcWRKY33A-F	TCTTCACTGTTGATACATATGATGGCTGCTTCTTCT	
pRI101-BcWRKY33A-R	GCTCACCATGGATCCGGTACCGGACAAAAACGAATC	
pTRV2-PDS-F	GTGAGTAAGGTTACCGAATTCTTCTGCGGCGAATTT	**Virus-induced gene silencing**
pTRV2-PDS-R	TCCCCATGGAGGCCTTCTAGAAGAAACTCTTAACCG	
pTRV2-MYB51-F	GTGAGTAAGGTTACCGAATTCAGCTCGTGGACTACC	
pTRV2-MYB51-R	TCCCCATGGAGGCCTTCTAGATTGACGTTCATAGAC	

Table S4 Recipes of V8 and SMB medium

**V8 agar medium**
72 ml V8 juice
4 g Agar
0.4 g $CaCO_3$
Add ddH_2O to 200 ml
Autoclave at 121 °C for 15 min
**SMB medium**
0.25 g Peptone
1 g Maltose
Add ddH_2O to 25 ml
Final pH (at 25 °C): 5.6 ± 0.2
Autoclave at 121 °C for 15 min

Frerigmann, H., & Gigolashvili, T. (2014). MYB34, MYB51, and MYB122 distinctly regulate indolic glucosinolate biosynthesis in Arabidopsis thaliana. *Molecular Plant*, 7(5), 814-828.
